# Editorial: Creation and utilization of crop germplasm resources

**DOI:** 10.3389/fpls.2023.1140037

**Published:** 2023-01-25

**Authors:** Chuanzhi Zhao, Sunil S. Gangurde, Xia Xin, Rajeev K. Varshney

**Affiliations:** ^1^ Institute of Crop Germplasm Resources (Institute of Biotechnology), Shandong Academy of Agricultural Sciences; Shandong Provincial Key Laboratory of Crop Genetic Improvement, Ecology and Physiology, Jinan, China; ^2^ College of Life Sciences, Shandong Normal University, Jinan, China; ^3^ Crop Protection and Management Research Unit, United States Department of Agriculture-Agricultural Research Service (USDA-ARS), Tifton, GA, United States; ^4^ Department of Plant Pathology, University of Georgia, Tifton, GA, United States; ^5^ National Crop GeneBank, Institute of Crop Sciences, Chinese Academy of Agricultural Sciences, Beijing, China; ^6^ State Agricultural Biotechnology Centre, Centre for Crop and Food Innovation, Food Futures Institute, Murdoch University, Murdoch, WA, Australia

**Keywords:** germplasms, mutants, gene identification, QTLs, molecular breeding

## Introduction

The world’s population will almost exceed 8 billion in 2022 (https://www.unfpa.org/swp2022). In contrast, the available area of cultivated land is decreasing every year. Therefore, it is a big challenge for international agriculture to ensure the food security of such a huge population (Pathirana and Carimi, 2022). With the recent COVID-2019 pandemic, global warming, economic slowdowns, poverty, and inequality have increased unemployment, resulted in food insecurity, and affected millions of people globally (FAO, 2021). The State of Food Security and Nutrition in the World 2021, https://www.fao.org/3/cb4474en/online/cb4474en.html). To address the increasing food demands, it is crucial to develop improved crop varieties with enhanced yield through the utilization of diverse germplasm resources (Varshney et al., 2021a).

Crop germplasm resources (CGRs) refer to the genetic material passed from parent to offspring of a crop, which is usually found in a specific variety. Diversity of germplasm is essential for improving multiple traits and increasing genetic gain. Due to the intensive production methods of modern agriculture, many local varieties of crops have been replaced by a few improved elite varieties. This has resulted in decreased genetic diversity in most of the crops. Several cultivated crops lost their important alleles due to selection during human civilization. Crops with a narrow genetic base make it difficult to breed new varieties with high yield and quality; for instance, peanut has resulted in a narrow genetic base during human civilization (Gangurde et al., 2019). Beneficial wild alleles associated with disease resistance and yield traits were lost during domestication and are needed today in modern breeding programs (Bohra et al., 2021; Varshney et al., 2021b). For instance, numerous such wild species from the genera *Aegilops*, *Agropyron*, *Amblyopyrum*, *Dasypyrum*, *Elymus*, *Leymus*, *Pascopyrum*, *Roegneria*, and *Thinopyrum* are growing in a wide range of areas and adapting to diverse climates, and most of them have been used for the genetic improvement of wheat (Soreng et al., 2015; Laugerotte et al., 2022). Similarly, *Haynaldia villosa* is a diploid wild grass with numerous traits similar to those of wheat, including high protein content, high resistance to cold, and resistance to powdery mildew (Cao et al., 2011). The wheat–*H. villosa* translocation line 6VS/6AL has been applied as the backbone parent of wheat breeding, resulting in the release of dozens of commercial cultivars in China, the USA, and Canada (Cao et al., 2011). As one of the most important oil crops worldwide, cultivated peanut is an allotetraploid, derived from hybridization between the progenitor species *A. duranensis* and *A. ipaensis* (Lavia et al., 2011; Kochert et al., 1996; Halward et al., 1992; Zhao et al., 2021). There are more than 80 species of the genus *Arachis* classified into nine sections, providing great potential to diversify the cultivated gene pool for peanut breeding. One of the main cultivated peanut cultivars, Yuanza9102, showed high resistance to *Ralstonia solanacearum* due to the introgression of the resistance gene of the wild diploid peanut species *Arachis diogoi* (Wang et al., 2018; Han et al., 2022). Therefore, collection, creation, evaluation, conservation, and utilization of germplasm resources, especially from wild species, can help improve the cultivated gene pool.

In 1920, a Soviet geneticist named Nicolai Vavilov realized that most crops had lost their diversity, and he created the first gene bank in Petrograd by doing an extensive series of expeditions worldwide (Janick, 2015). Later, several gene banks were developed in the USA, Western Europe, and Australia (Pathirana and Carimi, 2022). In 1991, the International Plant Genetic Resources Institute (IPGRI) signed an agreement in Rome, Italy, and developed the Crop Genetic Resources Collaboration Network to conserve and utilize germplasm resources. The network could collect ~200,000 accessions from 136 countries (Pathirana and Carimi, 2022). Utilization of these germplasm resources to enrich the cultivated crop varieties is a burning task for current breeding programs. Advances in genomics, phenomics, and bioinformatics have opened new frontiers for the conservation and utilization of germplasm resources. In our Research Topic entitled “creation and utilization of crop germplasm resources,” we published 17 research articles that shed light on the genetic diversity and evolution of crop germplasm resources, including wild relatives, landraces, and cultivated varieties, by using whole genome resequencing and association analysis, phenotyping, and Distinctness, Uniformity, and Stability (DUS) characterization, concerning ideotypes, tolerance to biotic/abiotic stresses, high yield, and nutritional traits. Furthermore, the included articles contributed to the identification of important genes/QTLs and uncovered the molecular mechanisms of those important traits.

## Germplasm characterization, genetic relationship, and population structure of collected germplasms

Germplasm characterization is very important to identify potential sources of traits of interest. For instance, 560 soybean cultivars, comprised of 279 cultivars from Northeast and Northwest China (NNC), 155 cultivars from the Huang-Huai-Hai Valleys (HHH), and 126 from Southern China (SC), were collected and designated as Modern Chinese Soybean Cultivars (MCSCs). Population structure analysis identified 13 maturity groups in soybeans in different ecoregions. The germplasms of NNC showed high allele diversity but were distant when compared with those of HHH and SC. Eleven major core-terminal ancestor-derived families, including four derived from ancestors in NNC, four from HHH, and three from SC, contain 463 (82.68%) MCSCs with some cross-distribution among ecoregions (Li et al.). Based on these findings, we can predict how important it is to exchange germplasm to enhance the genetic potential of crops.

Distinctiveness, uniformity, and stability (DUS) are the criteria for a new variety release for cultivation in a farmer’s field. A total of 195 wheat varieties from the Huang-Huai-Hai region of China were evaluated for 35 DUS characteristics. Of the 35 traits, eight characteristics varied significantly, with the most variation in the sterile spikelet. Artificial selection trends in flag leaf length and flag leaf width, grain number per ear, and grain volume weight showed an overall upward trend. In contrast, plant height showed a downward trend. These findings indicated that selection of some non-economic characteristics of wheat varieties, such as awn color, stem color, and glume color, seemed to be able to enrich the genetic diversity of varieties in the Huang-Huai-Hai region (Wang et al.).

The brewing industry in China is very popular, and Chinese sorghum has been an important ingredient in brewing since ancient times. Re-sequencing of 244 Chinese sorghum accessions resulted in three genetic clusters, namely the Northern, the Southern, and the Chishui groups. An important selective sweep region was identified with homologous genes involved in grain size, pericarp thickness, and the architecture of the inflorescence. These results also suggested that pericarp strength determines the ability of grain to resist repeated cooking during the brewing process (Zhang et al.).

The alleles from *in situ* conserved wild rice (*Oryza rufipogon* Griff.), also called “Guiping wild rice,” can improve rice production worldwide. A comparison of resequencing data between Guiping wild rice populations and *O. rufipogon* and *Oryza sativa* populations indicated that the *in situ* conserved wild rice population has a unique genetic structure, with genes that were introgressed from aromatic and *O. sativa* ssp. *indica* and *japonica* populations (Yang et al.).

## Genome-wide association study and multiple omics facilitated mining of beneficial genes/loci

The comparative metabolomics study of four peanut testa colors, including pink, purple, red, and white, identified 78 differentially accumulated metabolites. The proanthocyanidins are most abundant in pink testa, whereas, the red testa accumulated more isoflavones, flavonols, and anthocyanidins (Zhang et al.). Similarly, the integrated analysis of the metabolome and transcriptome identified 14 carotenoids in five sweet potato cultivars and 27 differentially expressed genes involved in carotenoid metabolism, respectively (Jia et al.). To identify candidate genes and allelic diagnostic markers for resistance to *R. solanacearum* of cultivated peanut, QTL-seq analysis identified the resistance gene mapped in a 7.2 Mb physical region of chromosome 12 of *Arachis hypogaea*, and eight nucleotide binding site leucine rich repeat genes were highlighted. Interestingly, two diagnostic SNP markers were developed and validated for breeding disease-resistant peanut varieties (Zhang et al.). Cold stress is an important abiotic stress affecting plant growth and development by interfering with physiological processes. A combinatorial approach to small RNA and degradome sequencing identified 407 known miRNAs and 143 novel peanut-specific miRNAs. Transient expression analysis in *Nicotiana benthamiana* showed that miR160, miR482, and miR2118 may play positive roles and that miR396; miR162; and miR1511 play negative roles in the regulation of peanut cold tolerance (Zhang et al.).

In this special issue, we also reported genetic dissection and candidate gene/locus identification for several abiotic stresses, such as salinity tolerance, nitrogen utilization efficiency, cold tolerance, and photoperiod response. Salinity–alkalinity is among the serious abiotic stresses limiting the yield potential of several crops. For instance, an association panel of 200 maize lines identified nine SNPs and associated candidate genes with alkaline tolerance in maize seedlings. RNA-Seq analysis confirmed five hub genes involved in alkaline tolerance (Li et al.). The salinity–alkalinity tolerance of rice varieties at the germination stage is very important because of the widespread use of direct seeding technology in rice. Based on the evaluation of seven germination-related traits on a 428-rice diversity panel, Xian/indica accessions showed generally higher tolerance to alkali stress than Geng/japonica accessions. Further, the GWAS identified 90 loci and eight candidate genes associated with the alkali tolerance. Interestingly, a negative regulator of alkali tolerance gene LOC_Os09g25060 (OsWRKY76) was identified (Mei et al.). Among two subspecies of rice, geng/japonica has significantly lower nitrogen-use efficiency (NUE) than xian/indica. Haplotype analysis based on 14 cloned genes of NUE and 36 rice germplasm lines developed 18 intragenic markers. The results reported 12 NUE genes, which are mostly present in XI accessions. The elite haplotype of gene *DEP1* is fixed in *geng/japonica* cultivars, and elite haplotypes of genes *MYB61* and *NGR5* have been introduced into some approved *geng/japonica* cultivars (Li et al.). Though foxtail millet is a model crop, it is not globally distributed and utilized due to its photoperiod sensitivity. Genetic mapping for photoperiod sensitivity using a RIL population (Longgu 3 × Canggu 3) identified 21 QTLs and 116 candidate genes. A candidate gene, *SiCOL5*, was identified as photoperiod-sensitive and regulated by biological rhythm-related genes (Li et al.), which might shed light on photoperiod-tolerant breeding.

## Identification of the function of candidate genes

Maize seeds are deficient in the essential amino acids cysteine (Cys) and methionine (Met). The improved highest Met maize line, pRbcS : AtSAT1-pRbcS : EcPAPR, increased Met by 2.24-fold. But the plants of pRbcS : AtSAT1-pRbcS : EcPAPR showed progressively severe defects in plant growth, including early senescence, stunting, and dwarfing, indicating that excessive sulfur assimilation has an adverse effect on plant development. The transcriptome analysis of maize leaves using pRbcS : AtSAT1-pRbcS : EcPAPR identified 3,274 differentially expressed genes associated with Met homeostasis. Two genes, serine/threonine–protein kinase (CCR3) and heat shock 70 kDa protein (HSP), were identified in the core of the leaves and endosperm, respectively (Xiang et al.). Isopentenyl transferase (IPT) is an important rate-limiting enzyme in cytokinin (CTK) synthesis in plants. In maize, over-expression of *ZmIPT2* led to delayed senescence of leaves and 17.71%–20.29% increases in grain yield, providing new insight for the breeding of new high-yield transgenic maize varieties (Song et al.).

## Creation and utilization of new germplasms by mutagenesis and introgression of foreign DNAs or chromosome fragments

Mutation breeding can help expand the genetic base by producing novel alleles with the help of chemical and physical mutagens. In field peas, physical and chemical mutagenesis were carried out using gamma irradiation and ethyl methanesulfonate (EMS), respectively. A gamma radiation dose of 225 Gy and an EMS concentration of 5 mm were selected as optimal dosages for mutagenesis in field peas. PEG-mediated transformation and gene editing of the LOX gene were carried out using the CRISPR/Cas system, providing the platform for creating new germplasms (Pandey et al.). *Thinopyrum intermedium* (JJJsJsStSt, 2n = 6x = 42), with good resistance to many wheat diseases, is known as one of the most important closely related wild species of wheat. A new line, CH51, was developed from the BC_1_F_8_ progeny of a partial wheat–*T. intermedium* amphiploid TAI8335 and wheat cultivar (cv.) Jintai 170. The CH51 showed high levels of resistance to the prevalent Chinese leaf rust and stripe rust races in the field and can be used to increase the resistance of wheat (Zhang et al.).

## Author contributions

CZ and SG drafted the manuscript. XX and RV provided input and comments to the draft. All authors listed have made a substantial, direct, and intellectual contribution to the work and approved it for publication.
